# The indirect effects of perfectionism on athletes’ self-views through maladaptive emotion regulation

**DOI:** 10.3389/fpsyg.2024.1373461

**Published:** 2024-06-24

**Authors:** Hollie Minichiello, Madisen Reasonover, Paul Fuglestad

**Affiliations:** Department of Psychology, University of North Florida, Jacksonville, FL, United States

**Keywords:** perfectionism, emotion regulation, self-esteem, athletics, athletes

## Abstract

**Introduction:**

In general, increased levels of perfectionism have been associated with increased levels of burnout, heightened levels of depression and anxiety, lowered self-esteem, and poorer overall performance, yet perfectionistic strivings within athletes have also been associated with lower burnout and better performance in some contexts.

**Methods:**

The current study investigated whether emotion regulation strategies would indirectly link perfectionism with self-esteem in young adults who had participated in competitive athletics. Two hundred and fifty-three primarily white (60.0 %), female (83.0 %) undergraduate students who had participated in competitive athletics completed a series of questionnaires including: the Self-liking and Self-Competence Scale – Revised, the Cognitive Emotion Regulation Questionnaire, and the short form of the Multidimensional Perfectionism Scale.

**Results:**

The PROCESS macro for SPSS was used to examine the indirect association between perfectionism and self-esteem through emotion regulation. Higher self-oriented perfectionism and socially prescribed perfectionism were both indirectly associated with lower self-liking and self-competence through greater catastrophizing and self-blame.

**Discussion:**

For individuals like athletes, who experience internal and external pressures, increased perfectionism may lead to negative self-views through maladaptive emotion regulation. However, longitudinal and experimental work is needed to establish this proposed pattern of relationships.

## Introduction

Approximately eight million people in the United States, high school age and above, engage in competitive athletics (Schwarb, 2018). With the pressure put on athletes’ performance, there is potential for negative outcomes in these individuals (Young et al., 2015). Previous research with athletes has shown that higher perfectionism is linked to lower self-esteem, decreased motivation, and increased burnout (e.g., Gould, 1996; Gotwals et al., 2003; *cf.* Madigan et al., 2016). Further, emotion regulation techniques, like displacement of anger, self-blame, and catastrophizing, can occur due to increases in self-oriented and socially prescribed perfectionism (Haase et al., 2002). Given the potential of negative outcomes due to perfectionism, the current study investigated the psychological pathways from perfectionism to two facets of self-esteem—self liking and self-competence—through adaptive and maladaptive emotion regulation techniques. Sociometer theory (Leary and Baumeister, 2000; Reitz et al., 2016), which proposes that self-esteem operates as an internal meter influenced by relationships and social standing, served as a theoretical framework linking the key constructs of the present study.

### Self-esteem

Self-esteem is one’s overall attitude or evaluation of oneself (Leary and Baumeister, 2000; Reitz et al., 2016). Research has shown that an individual’s behavior can change due to the perception of the self, in that individuals may seek validation for their feelings toward themselves from others (Leary et al., 1995; De Cuyper et al., 2013). This can be particularly harmful to athletes, who already set high standards for themselves (Tafarodi and Swann, 1995; Hill et al., 2010). Similarly, sociometer theory proposes that self-esteem is the assessment of an individual’s behavior promoting social inclusion while minimizing exclusion within a context of external social stimuli (Leary, 1990).

Diggory (1966) suggests that we base our self-evaluation on two broad criteria: social approval/acceptance and evaluation of abilities. In line with this, researchers have considered social worth and competence as facets of self-esteem (Tafarodi and Swann, 1995). Social worth, referred to as self-liking in the current study, emphasizes positive regard from other individuals, which in turn, causes the individual to view themselves as acceptable or unacceptable. Thus, self-liking is a socially dependent source of self-esteem (Tafarodi and Swann, 1995). Competence, referred to as self-competence in the current study, acknowledges the individual’s need for successful actions. This results from successful manipulation of the environment and external stimuli to promote goal achievement. Thus, if the individual has higher self-competence, they view themselves as highly capable, effective, and in control (Tafarodi and Swann, 1995). Both of these facets of self-esteem should be relevant to perfectionism.

### Perfectionism

Perfectionism can be conceptualized as extremely critical evaluations of the self, paired with high standards of performance (Frost et al., 1990; Hewitt et al., 1991; Flett and Hewitt, 2002). In one well-established model, perfectionism is broken down into three dimensions—self-oriented, other-oriented, and socially prescribed perfectionism. Self-oriented perfectionism relates to high expectations for success, which are driven by internal beliefs that align with the idea that being perfect is important (Hewitt and Flett, 1991; Stoeber, 2014). Self-oriented perfectionism has been linked to higher levels of self-esteem, conscientiousness, goal attainment, and positive affect, compared to other dimensions of perfectionism (Powers et al., 2005; Molnar et al., 2006; Trumpeter et al., 2006). This may be due to the intrinsic motivation relevant to self-oriented perfectionism (Mills and Blankstein, 2000; Stoeber et al., 2009). However, in a meta-analysis of perfectionism and mental health (Limburg et al., 2017), self-oriented perfectionism was associated with a number of psychological disorders and symptoms (e.g., anorexia, bulimia, obsessive beliefs, worry).

Socially prescribed perfectionism is the perceived desire for validation from others paired with interpersonal sensitivity and belief that others are imposing unrealistic expectations (Hewitt and Flett, 1991; Hill et al., 2010; Curran and Hill, 2018). Socially prescribed perfectionism has been linked to neuroticism, negative affect, and psychological disorders and symptoms (Molnar et al., 2006; Stoeber et al., 2009; Limburg et al., 2017). Since socially prescribed perfectionists strive for flawlessness and an ideal public self-image, these individuals are at higher risk for developing their ideal self with the public in mind, which may promote a precarious self-image (Hewitt and Genest, 1990).

Due to the focus on expectations in others and not the self (i.e., expecting others to be perfect), other-oriented perfectionism was not examined in the current study.

### Perfectionism in athletics

Athletes may experience external pressures from coaches and parents, which potentially reinforces the development and maintenance of perfectionism (Mageau and Vallerand, 2003; Fleming et al., 2022). With the pressure put on athletes to perform, there is potential for negative outcomes. Research has shown that certain aspects of perfectionism (e.g., parental criticism and concern over mistakes) are associated with lower self-esteem (Gotwals et al., 2003). Further, athletes who score higher in perfectionism are at higher risk for decreased motivation, which can lead to higher levels of burnout (Gould, 1996; Crowell and Madigan, 2022). Thus, high achieving individuals can become emotionally and physically exhausted because they must do something they may no longer feel like doing. The exhaustion caused by this may be stressful and increase feelings of anxiousness (Hill and Curran, 2015). These stressful events may evoke unhealthy coping mechanisms, such as increased risk of eating disorders and emotion regulation techniques such as displacement of anger or denial (Haase et al., 2002). Contrasting this, other models of perfectionism (e.g., personal standards perfectionism) have been shown to buffer feelings of emotional and physical exhaustion in athletes (Madigan et al., 2016; Stoeber and Madigan, 2016). Because the role of perfectionism in athletes is complex, it is important to consider other psychological constructs such as emotion regulation, which may account for associations between perfectionism and self-esteem.

### Emotion regulation

Proper emotion regulation is an important factor in the overall well-being of the athlete. Emotion regulation refers to the ways we evaluate, monitor, and modify emotional experiences (Thompson, 1994; Gross and Thompson, 2007). Further, emotion regulation involves assessing the intensity of emotional behaviors and the features they present to reach a set goal (Thompson, 1994). Within emotion regulation, there are two broad techniques: cognitive reappraisal, which is considered adaptive, and expressive suppression, which is considered maladaptive. Cognitive reappraisal is changing the way one thinks about an emotional situation, while expressive suppression is changing the behavioral response to an ongoing emotional event (Lazarus and Alfert, 1964; John and Gross, 2004). Likewise, cognitive reappraisal consists of emotion regulation strategies such as refocusing on planning, positive reappraisal, putting into perspective, and acceptance. Contrasting this, expressive suppression consists of emotion regulation strategies such as self-blame, rumination, and catastrophizing (Garnefski and Kraaij, 2006). Further differentiating the two techniques, cognitive reappraisal occurs earlier in the emotion-generative process, while expressive suppression occurs later (Gross and John, 2003). Therefore, cognitive reappraisal allows the individual to modify their behavioral expressions of emotions as well as their internal experience of emotions. Contrasting this, expressive suppression only modifies what the individual expresses behaviorally because of emotions, and thus may be costly to the individual’s psychological well-being (Gross and John, 2003).

The process model of emotion regulation is the most frequently used emotion regulation framework (Gross and Thompson, 2007). In this model, individuals assess, attend to, reappraise, and respond to emotion-evoking events. The process begins with situation selection, which involves assessing whether we think a situation will evoke the emotions we would like to experience or decrease the likelihood of emotions we do not want. When a situation occurs, we modify our behaviors to avoid unwanted emotions. Situation modification involves directly modifying the emotion-evoking event to change its impact on our emotions. In responding to an emotion-evoking situation, we may use cognitive reappraisal techniques to change the way we think about the situation, or we may use expressive suppression techniques to inhibit our emotional expressions or behaviors. Once emotion regulation techniques have modified the situation, the process restarts upon the next encounter of an emotion-evoking situation.

Emotion regulation techniques are developed throughout childhood when many individuals are engaging in rigorous athletics and setting personal educational standards (Vois and Damian, 2020). In childhood, individuals rely more on expressive suppression than cognitive reappraisal, which influences emotional reactions during competitive sports. As people age, reliance upon expressive suppression decreases while cognitive reappraisal increases (Gross and Levenson, 1993). However, emotion regulation strategies (i.e., cognitive reappraisal and expressive suppression) in response to emotion-evoking situations like competitive sports can be problematic when combined with other person characteristics like perfectionism.

Perfectionism can lead to maladaptive emotion regulation techniques, such as self-blame and rumination (Rudolph et al., 2007; Macedo et al., 2017). High levels of perfectionism have also been shown to cause psychological distress, which in turn produces disruptions to emotion regulation (Macedo et al., 2017). Likewise, individuals with high personal standards and high evaluative concerns tend to score higher in expressive suppression versus cognitive reappraisal, engaging in techniques like catastrophizing and rumination (Hill and Davis, 2014). This is problematic in that expressive suppression works to decrease the behavioral responses of negative emotions, not the internal experience of negative emotions, and may also suppress positive emotions (Gross and John, 2003).

### Sociometer theory

Sociometer theory provides a useful theoretical framework for understanding the interrelations among perfectionism, emotion regulation, and self-esteem. The first phase of the theory begins with an individual’s perception of social exclusion. This may be relevant to self-oriented perfectionism, in which the individual does not view themselves as good enough to be relevant to a social group. Additionally, this phase may relate to socially prescribed perfectionism, in that the individual perceives that the social group expects them to be perfect and they are not living up to such expectations (Dunkley et al., 2012). In this phase, an athlete may perceive signs of social exclusion from individuals such as coaches. Due to social exclusion cues, the individual’s self-esteem is diminished. This represents phase two of sociometer theory and potentially connects to decreased self-liking and self-competence within the individual.

Koivula and Hassme (2002) found that athletes whose self-esteem was dependent on self-competence displayed more negative perfectionism (e.g., concern over mistakes, doubts about actions) than athletes whose self-esteem was based on aspects like self-respect and self-love. Similar research has shown associations between perfectionism (e.g., parental criticism, concern over mistakes) and decreased self-esteem (Gotwals et al., 2003). In reaction to decreased self-esteem, the individual then enters phase three, in which they experience aversive emotions. This phase may reflect emotion regulation techniques, such as rumination and self-blame (Gold and Wegner, 1995; Martin and Tesser, 1996; Orth et al., 2006). In the last phase of sociometer theory, the individual applies re-inclusion behaviors to diminish signs of social exclusion and solidify their standing as a member of the social group. Regarding athletes, examples of re-inclusion behaviors may include working harder in practice and competitions to please the individual or social group that initially displayed signs of exclusion.

Given the interconnections between perfectionism, emotion regulation, and self-esteem, there are potential implications for athletes. Because dimensions of perfectionism can be maladaptive, perfectionistic tendencies can threaten the way the individual views themself. This, in turn, can decrease self-confidence and increase general anxiety, competence anxiety, and depression in athletes (Koivula and Hassme, 2002; Macedo et al., 2017). However, if athletes are able to rely on adaptive emotion regulation techniques, such as refocusing on planning, acceptance, and putting things into perspective, they may be able to not only buffer decreases in self-esteem, but potentially boost their self-esteem (Rice et al., 1998; Koivula and Hassme, 2002).

### Study overview

The current study examined the associations between perfectionism, emotion regulation, and self-esteem within athletes. Athletes are represented by a sample of college students who participated in competitive athletics for at least three years as adolescents. More specifically, the study explored the indirect links of maladaptive and adaptive emotion regulation in the associations between self-oriented and social prescribed perfectionism and self-liking and self-competence.

The current research investigated two interrelated research questions:

1. Are self-oriented and socially prescribed perfectionism associated with poor emotion regulation and lower self-esteem? It was hypothesised that self-oriented and socially prescribed perfectionism would be associated with poor emotion regulation and lower self-esteem.
2. Is emotion regulation a potential link between self-oriented and socially prescribed perfectionism and self-esteem? It was hypothesised that maladaptive emotion regulation would indirectly link self-oriented and socially prescribed perfectionism with self-liking and self-competence.

## Method

### Participants

The initial sample consisted of the 258 athletes, with ages ranging from 18–49 years old and the majority identifying as white (*n* = 181). Thirty-six participants reported being biologically male at birth and 212 reported being biologically female at birth (10 did not indicate sex). All individuals were undergraduate students at a mid-sized university in the Southeastern United States (Table 1 displays participant characteristics). To qualify for inclusion, participants had to have participated in competitive athletics for at least three years. The most common sports were soccer (44), basketball (28), track/cross country (23), softball/baseball (22), swimming (21), volleyball (20), dance (17), cheer (15), and gymnastics (9). The pwr2ppl package in R was used to estimate the power to detect the proposed indirect effects of perfectionism with self-esteem through emotion regulation. Based on correlations from previous literature between study variables (e.g., *r’s* in the magnitude of 0.25 to 0.40 between perfectionism, maladaptive emotion regulation, and self-esteem; Gotwals et al., 2003; Rudolph et al., 2007; Macedo et al., 2017), a sample of 150 was estimated to give adequate power (0.80) to detect indirect effects (e.g., socially prescribed perfectionism → maladaptive emotion regulation → self-liking). Full details of the power analysis can be found in the project OSF page.^1^

**Table 1:** Participant demographic characteristics.

	*N* = 258
Age (years)	20.63 ± 4.31; range: 18–49
Sex Assigned at Birth	
Female	212 (85.5%)
Male	36 (14.5%)
Intersex	0 (0.0%)
Race/Ethnicity	
White/Caucasian	181 (65.57%)
Black/African American	36 (13.04%)
Hispanic/Latino	39 (14.13%)
Asian	9 (3.26%)
Pacific Islander	0 (0.0%)
Middle Eastern	2 (0.72%)
Native American	3 (1.08%)
Multiple Races	5 (1.81%)
Other	1 (0.36%)
Household income	
Under $25,000	45 (18.5%)
$25,000 - $39,999	37 (15.2%)
$40,000 - $49,999	25 (10.3%)
$50,000 - $79,999	40 (16.5%)
$75,000 - $99,999	35 (14.4%)
$100,000 - $149,999	30 (12.3%)
Over $150,000	31 (12.8%)

Counts and percentages are given for nominal variables. Means and standard deviation are given for continuous variables.

### Procedure

The materials and procedures were approved by the university’s Institutional Review Board. Prior to data collection, participants reviewed and agreed to an electronic informed consent. Per the APA Ethical Principles of Psychologists and Code of Conduct, participant treatment was ethical with maximization of benefits and minimization of risks (American Psychological Association, 2017). After confirming consent, participants completed questionnaires, which were presented through block randomization via qualtrics. The participants ended the survey by completing demographic questions. Participants were compensated with extra credit for their classes through the university’s psychology recruitment system.

### Materials

#### Perfectionism

Participants completed the short form version of the Multidimensional Perfectionism Scale (Hewitt et al., 1991). This 15-item, self-report scale was developed to assess three perfectionism subscales: self-oriented (*α* = 0.69), other-oriented (*α* = 0.66), and socially prescribed perfectionism (*α* = 0.77). The participants were asked to indicate agreement with each statement using a 1 (*strongly disagree*) to 7 (*strongly agree*) scale. Each subscale score was computed by summing all items relevant to each subscale. Previous research supports the validity of this scale in young adults, adulthood, and college students both male and female (Hewitt et al., 1991).

#### Emotion regulation

Participants completed the Cognitive Emotion Regulation Questionnaire-short form (Garnefski and Kraaij, 2006). This 18-item, self-report questionnaire was designed to assess nine aspects of emotion regulation based on different scenarios (two items for each aspect). The scenario given for each participant was to think of a time when they were disappointed. Participants were then asked to indicate agreement with each statement using a 1 (*strongly disagree*) to 5 (*strongly agree*) scale. The nine different aspects are catastrophizing (*α* = 0.92), self-blame (*α* = 0.91), rumination (*α* = 0.78), blaming others (*α* = 0.82), acceptance (*α* = 0.82), positive refocusing (*α* = 0.83), planning (*α* = 0.66), positive reappraisal (*α* = 0.84), and putting into perspective (*α* = 0.67). Scores for each aspect were calculated by summing the two items. Previous research supports adequate validity of this questionnaire in both men and women aged 18 and older (Garnefski and Kraaij, 2006).

#### Self-liking and self-competence

Participants completed the Self-Liking and Self-Competence Scale-Revised (Tafarodi and Swann, 2001). This 16-item, self-report scale was designed to assess two aspects of global self-esteem: self-liking (*α* = 0.92) and self-competence (*α* = 0.74). Participants were asked to indicate agreement with each of the statements using a 1 (*strongly disagree*) to 5 (*strongly agree*) scale. Each aspect was computed by summing all relevant items. Previous research provides evidence for the convergent and discriminant validity of this questionnaire in adults aged 18 and older (Tafarodi and Swann, 2001).

## Results

### Correlational analysis

Table 2 shows bivariate correlations among all study variables. Self-oriented perfectionism was positively related to catastrophizing, self-blame, and rumination, but negatively related to self-liking. Socially prescribed perfectionism was positively related to catastrophizing, self-blame, rumination, and other blame, but negatively related to positive reappraisal and self-liking. Self-liking was negatively related to catastrophizing, self-blame, and rumination, but positively related to positive refocusing, positive reappraisal, and putting into perspective. Self-competence was negatively related to catastrophizing, self-blame, and rumination, but positively related to positive reappraisal and putting into perspective.

**Table 2:** Bivariate correlations of perfectionism, emotion regulation, and self-esteem.

	1.	2.	3.	4.	5.	6.	7.	8.	9.	10.	11.	12.	13.
1.Self-Oriented Perfectionism	–												
2.Socially Prescribed Perfectionism	0.41^***^	–											
3.Catastrophizing	0.29^***^	0.31^***^	–										
4.Self-blame	0.26^***^	0.19^**^	0.41^***^	–									
5.Rumination	0.14^*^	0.22^***^	0.47^***^	0.31^***^	–								
6.Other Blame	−0.05	0.14^*^	0.09	−0.36^***^	0.03	–							
7.Acceptance	−0.07	−0.08	−0.04	0.08	0.18^**^	0.03	–						
8.Refocusing	−0.03	0.03	−0.18^**^	−0.16^*^	−0.05	19^**^	0.14^*^	–					
9.Planning	0.11	−0.09	0.09	0.20^**^	0.16^*^	−0.13^*^	0.21^***^	0.06	–				
10.Reappraisal	−0.09	−0.15^*^	−0.16^*^	−0.06	−0.05	−0.06	0.42^***^	0.16^*^	0.32^***^	–			
11.Perspective	−0.02	−0.06	−0.17^**^	0.11	−0.05	0.003	0.19^**^	0.17^**^	0.21^**^	0.22^***^	–		
12.Self-Liking	−0.17^**^	−0.27^***^	−0.50^***^	−0.33^***^	−0.30^***^	−0.002	0.06	0.16^*^	0.001	0.23^***^	0.18^**^	–	
13.Self-Competence	0.05	−0.08	−0.36^***^	−0.28^***^	−0.20^**^	0.03	0.06	0.01	0.08	0.23^***^	0.15^*^	0.68^***^	–
*M*	25.86	21.26	7.01	6.94	7.56	4.75	8.18	5.90	7.88	8.70	6.92	22.97	23.80
*SD*	4.98	5.78	2.35	2.35	1.95	2.03	1.69	2.21	1.70	1.51	1.94	8.08	4.79

^*^p < 0.05, ^**^p < 0.01, ^***^p < 0.001.

### Mediation analyses

The PROCESS Macro for SPSS (Hayes, 2022) was used to examine the indirect effects of emotion regulation in the associations between perfectionism and self-esteem. More specifically, self-oriented perfectionism and socially prescribed perfectionism were each examined as predictors, and self-liking and self-competence were each examined as outcomes. The nine emotion regulation strategies were examined as mediators in each analysis. All variables were standardized prior to analysis. Table 3 shows all path coefficients and indirect effects. Full output and analysis details can be found on the OSF page.

**Table 3:** Pathway coefficients and indirect effects of the association of perfectionism with self-esteem through emotion regulation strategies.

	Socially prescribed perfectionism		Self-oriented perfectionism	
	β	Indirect effect	β	Indirect effect
Pathways: from perfectionism to emotion regulation				
Catastrophizing	**0.31 [0.19, 0.43]**		**0.29 [0.17, 0.41]**	
Self-blame	**0.19 [0.06, 0.31]**		**0.26 [0.14, 0.38]**	
Rumination	**0.22 [0.10, 0.34]**		**0.14 [0.02, 0.26]**	
Other Blame	**0.14 [0.01, 0.26]**		−0.05 [−0.17, 0.08]	
Acceptance	−0.08 [−0.20, 0.05]		−0.06 [−0.19, 0.06]	
Refocusing	0.03 [−0.10, 0.15]		−0.03 [−0.16, 0.10]	
Planning	−0.09 [−0.22, 0.03]		0.11 [−0.02, 0.23]	
Reappraisal	**−0.15 [−0.27, −0.02]**		−0.09 [−0.21, 0.04]	
Perspective	−0.05 [−0.18, 0.07]		−0.02 [−0.14, 0.11]	
Pathways: from emotion regulation to self-liking				
Catastrophizing	**−0.34 [−0.48, −0.20]**	**−0.11 [−0.17, −0.05]**	**−0.36 [−0.50, −0.22]**	**−0.10 [−0.18, −0.05]**
Self-blame	**−0.14 [−0.28, −0.005]**	−0.03 [−0.06, 0.0001]	**−0.16 [−0.30, −0.02]**	**−0.04 [−0.09, −0.01]**
Rumination	−0.06 [−0.18, 0.07]	−0.01 [−0.05, 0.02]	−0.07 [−0.20, 0.06]	−0.01 [−0.04, 0.01]
Other blame	−0.004 [−0.13, 0.12]	−0.001 [−0.02, 0.02]	−0.02 [−0.14, 0.10]	0.001 [−0.01, 0.02]
Acceptance	−0.01 [−0.13, 0.12]	0.001 [−0.01, 0.01]	0.001 [−0.12, 0.12]	0.000 [−0.02, 0.01]
Refocusing	0.04 [−0.07, 0.15]	0.001 [−0.01, 0.01]	0.03 [−0.08, 0.14]	−0.0001 [−0.02, 0.01]
Planning	−0.004 [−0.12, 0.12]	0.0003 [−0.01, 0.02]	0.01 [−0.11, 0.13]	−0.001 [−0.02, 0.02]
Reappraisal	0.12 [−0.004, 0.25]	−0.02 [−0.05, 0.003]	0.13 [0.00, 0.25]	−0.01 [−0.04, 0.005]
Perspective	0.10 [−0.02, 0.21]	−0.01 [−0.03, 0.01]	0.10 [−0.02, 0.22]	−0.002 [−0.02, 0.01]
Pathways: from emotion regulation to self-competence				
Catastrophizing	**−0.27 [−0.42, −0.12]**	**−0.08 [−0.15, −0.03]**	**−0.30 [−0.45, −0.15]**	**−0.09 [−0.16, −0.03]**
Self-blame	**−0.21 [−0.36, −0.06]**	**−0.04 [−0.08, −0.01]**	**−0.22 [−0.37, −0.08]**	**−0.06 [−0.11, −0.02]**
Rumination	−0.05 [−0.19,0.09]	−0.01 [−0.05,0.03]	−0.03 [−0.17,0.10]	−0.005 [−0.03,0.02]
Other Blame	0.01 [−0.12, 0.15]	0.002 [−0.02, 0.03]	0.03 [−0.10, 0.16]	−0.001 [−0.01, 0.01]
Acceptance	−0.01 [−0.15, 0.12]	0.001 [−0.01, 0.02]	−0.01 [−0.14, 0.12]	0.0004 [−0.01, 0.01]
Refocusing	**−0.13 [−0.26, 0.01]**	−0.004 [−0.03, 0.02]	**−0.13 [−0.25, −0.01]**	0.004 [−0.01, 0.03]
Planning	0.11 [−0.02, 0.24]	-0.01 [−0.04, 0.01]	0.08 [−0.04, 0.21]	0.01 [−0.01, 0.03]
Reappraisal	0**.16 [0.02, 0.29]**	**−0.02 [−0.06, −0.001]**	0**.16 [0.03, 0.30]**	−0.01 [−0.05, 0.01]
Perspective	0.09 [−0.03, 0.21]	−0.005 [−0.03, 0.01]	0.09 [−0.03, 0.21]	−0.002 [−0.02, 0.01]

Effects are standardized. 95% CIs are given in brackets. Effects in bold are considered significant.

#### Self-oriented perfectionism and self-liking and self-competence

**Figure 1:**
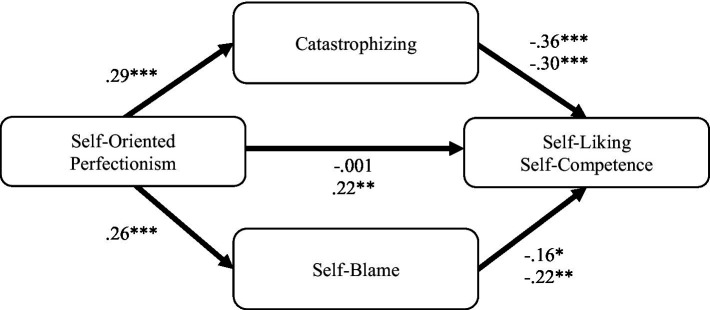
Parallel mediation pathways of self-oriented perfectionism to self-liking and self-competence. Path coefficients are standardized. Top coefficients correspond to self-liking as the outcome variable; bottom coefficients correspond to self-competence. ^*^p < 0.05, ^**^p < 0.01, ^***^p < 0.001.

Self-oriented perfectionism was indirectly associated with self-liking through catastrophizing and self-blame (Figure 1). Specifically, higher self-oriented perfectionism was associated with higher catastrophizing, *β = 0*.29, 95% CI [0.17, 0.41], and self-blame, *β = 0*.26, 95% CI [0.14, 0.38], which in turn were associated with lower self-liking, *β =* −0.36, 95% CI [−0.50, −0.22], *β =* −0.16, 95% CI [−0.30, −0.02]. The indirect effect through catastrophizing was −0.10, 95% CI [−0.18, −0.05], and the indirect effect through self-blame was −0.04, 95% CI [−0.09, −0.01].

Self-oriented perfectionism was also indirectly associated with self-competence through catastrophizing and self-blame (Figure 1). Specifically, higher self-oriented perfectionism was associated with higher catastrophizing, *β = 0*.29, 95% CI [0.17, 0.41], and self-blame, *β = 0*.26, 95% CI [0.14, 0.38], which in turn were associated with lower self-competence *β =* −0.30, 95% CI [−0.45, −0.15], *β =* −0.22, 95% CI [−0.37, −0.08]. The indirect effect through catastrophizing was −0.09, 95% CI [−0.16, −0.03], and the indirect effect through self-blame was −0.06, 95% CI [−0.11, −0.02]. There was also a positive direct effect of self-oriented perfectionism with self-competence, *β = 0*.21, 95% CI [0.09, 0.33].

#### Socially prescribed perfectionism and self-liking and self-competence

**Figure 2:**
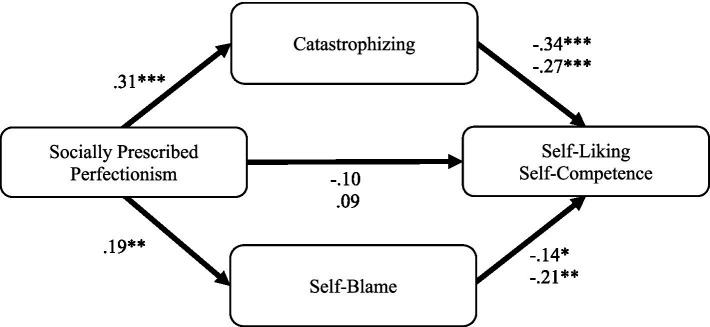
Parallel mediation pathways of socially prescribed perfectionism to self-liking and self-competence. Path coefficients are standardized. Top coefficients correspond to self-liking as the outcome variable; bottom coefficients correspond to self-competence. ^*^p < 0.05, ^**^p < 0.01, ^***^p < 0.001.

Socially prescribed perfectionism was indirectly associated with self-liking through catastrophizing (Figure 2). Specifically, higher socially prescribed perfectionism was associated with higher catastrophizing, *β = 0*.31, 95% CI [0.19, 0.43], which in turn was associated with lower self-liking, *β =* −0.34, 95% CI [−0.48, −0.20]. The indirect effect through catastrophizing was −0.11, 95% CI [−0.17, −0.05].

Socially prescribed perfectionism was indirectly associated with self-competence through catastrophizing and self-blame (Figure 2). Specifically, higher socially prescribed perfectionism was associated with higher catastrophizing, *β = 0*.31, 95% CI [0.19, 0.43], and self-blame, *β = 0*.19, 95% CI [0.06, 0.31], which in turn were associated with lower self-competence *β =* −0.27, 95% CI [−0.42, −0.12], *β =* −0.21, 95% CI [−0.36, −0.06]. The indirect effect through catastrophizing was −0.08, 95% CI [−0.15, −0.03], and the indirect effect through self-blame was −0.04, 95% CI [−0.08, −0.01].

## Discussion

The current study’s objective was to investigate the associations between perfectionism, emotion regulation, and self-esteem in athletes. Initial correlations between all study variables were conducted to assess the patterns of association. Dimensions of perfectionism—self-oriented and socially prescribed—were both correlated with maladaptive emotion regulation techniques and the self-liking subscale of self-esteem. Thus, in line with prior research, as perfectionism increased, so did reliance upon maladaptive emotion regulation techniques, while self-liking decreased (Vois and Damian, 2020; Kahn et al., 2021). Contrasting this, dimensions of perfectionism were unrelated to adaptive emotion regulation techniques and to the self-competence subscale of self-esteem.

The mediational analyses addressed the pathways from self-oriented perfectionism to self-liking and self-competence through adaptive and maladaptive emotion regulation techniques. In these models, indirect associations occurred from self-oriented perfectionism to self-liking and self-competence through maladaptive, but not adaptive, emotion regulation. Thus, increases in self-oriented perfectionism were associated with increases in the maladaptive emotion regulation techniques of catastrophizing and self-blame, which in turn were associated with decreases in self-liking and self-competence. Similar results were found in that maladaptive, but not adaptive, emotion regulation linked the associations of socially prescribed perfectionism with self-liking and self-competence. More specifically, increases in socially prescribed perfectionism were associated with increases in the maladaptive emotion regulation techniques of catastrophizing and self-blame, which in turn were associated with decreases in self-liking and self-competence. Overall, these results support the current study’s hypotheses as well as findings of prior research.

Research has demonstrated that higher levels of perfectionism—both self-oriented and socially prescribed—are related to lower levels of self-esteem and higher levels of psychological disorders and symptoms (Hewitt et al., 1994; Gotwals et al., 2003; Limburg et al., 2017). Further, research has shown that perfectionistic concerns are related to reliance upon maladaptive emotion regulation techniques, such as self-blame and rumination (Rudolph et al., 2007; Macedo et al., 2017). From the perspective of sociometer theory, negative associations between socially prescribed perfectionism and self-liking and self-competence could be due to the negative influence of external social stimuli such as peers, coaches, and parents. Those higher in socially prescribed perfectionism may also be more likely to interpret critiques from others as signs of social exclusion. As a result, these social influences may deplete individuals’ internal meters of self-esteem via the use of maladaptive emotion regulation techniques such as catastrophizing and self-blame, increasing the experience of maladaptive, aversive emotions (Leary and Baumeister, 2000). Thus, the current study’s findings in conjunction with prior research, provides evidence that both self-oriented and socially prescribed perfectionism are linked to poorer emotion regulation and lower self-esteem.

Although these findings are generally consistent with prior work, some research does suggest inconsistencies. For example, Madigan et al. (2016) demonstrated that evaluative concerns perfectionism predicted greater burnout and exhaustion over the course of 3 months, whereas personal standards perfectionism was protective with respect to burnout and exhaustion over the course of 3 months. Although not in the context of athletics, Macedo et al. (2017) found that evaluative concerns, but not strivings, were related to emotion regulation strategies (e.g., catastrophizing, rumination) and psychological well-being (anxiety and depression). The findings with respect to evaluative concerns are consistent with socially prescribed perfectionism in the present study. However, the effects of personal standards/strivings are at odds with the effects of self-oriented perfectionism in the present study in that self-oriented perfectionism indirectly predicted lower self-liking and self-competence through maladaptive emotion regulation strategies. At the same time, and more consistent with Madigan et al. (2016), there was also a direct, positive association between self-oriented perfectionism and self-competence (when controlling for emotion regulation). Although perfectionism theories and measurement frameworks are distinct, self-oriented perfectionism is similar to personal standards/striving and socially prescribed perfectionism is similar to evaluative concerns. Future research would benefit from a comparison of these two perfectionism models in the context of emotion regulation and personal well-being for athletes. It is possible that certain aspects of perfectionism (personal standards) may not lead to burnout but may still have pernicious effects on emotional regulation and psychological well-being over time.

### Implications

Individuals who participate in athletics may face additional stressors, with potentially maladaptive outcomes (Ong and Cheong, 2009). The lasting impacts of prolonged pressure from individuals surrounding the athlete can increase feelings of anxiety and negative affect (Molnar et al., 2006; Stoeber et al., 2009; Limburg et al., 2017). These stressful events may evoke maladaptive emotion regulation techniques, such as self-blame, catastrophizing, and displacement of anger (Haase et al., 2002; John and Gross, 2004). Further, individuals with poorer emotion regulation techniques are at higher risk for maladaptive outcomes, like lower self-esteem, according to our research. Thus, individuals, including athletes, should be encouraged to minimize their engagement with maladaptive techniques, like catastrophizing and self-blame. Rather, the promotion of adaptive strategies, like positive reappraisal and putting things into perspective, can work to increase positive emotions, decrease negative emotions, and buffer associations between negative events with maladaptive outcomes (Doorley and Kashdan, 2021). Given that many individuals with socially prescribed perfectionism create their ideal image of the self with the public in mind, it is imperative that these individuals have proper emotion regulation techniques to buffer the negative feelings they may experience (Hewitt and Genest, 1990).

Since athletes are often under performance and competitive pressure, it is important that athletic organizations work to minimize additional, extraneous pressures and exclusionary events. Additionally, organizations, coaches, and parents can promote and train athletes with personal skills, such as specific sport-related training and mental training, which can promote adaptive perfectionism and positive emotion regulation, further protecting self-esteem (Gonzalez-Hernandez et al., 2021). This is especially important for younger athletes as individuals rely more on expressive suppression than cognitive reappraisal in childhood (Gross and Levenson, 1993). It would be beneficial for parents and coaches to address adaptive emotion regulation techniques actively and explicitly. By intervening early, athletes can be equipped with the skills to handle future pressures and promote better psychological well-being (Madigan et al., 2019; Vois and Damian, 2020). Also, given the maladaptive associations observed in the present study and other research (Mageau and Vallerand, 2003; Fleming et al., 2022), coaches and parents should work to avoid instilling socially prescribed perfectionism through controlling behaviours and external pressures, but instead work to build feelings of relatedness, competence, and autonomy (Ryan and Deci, 2002; Bartholomew et al., 2009).

### Limitations

Although the study provided evidence for the indirect effects of maladaptive emotion regulation, it is important to note several limitations. To begin, all data were collected via self-report, which may impact the validity and reliability of the findings (Gregorich, 2006). In particular, bias could have occurred with respect to retrospective reports of emotional regulation and athletic involvement. Further, the study used a convenience sample at a mid-sized Southeastern university in the United States, which may not be generalizable (Heckman, 2010). Additionally, these are individuals who participated in competitive sports for a minimum of three years but may no longer participate in the same sport or sports in general. While all undergraduate students at this university were able to participate, the ones who did participate were able to receive extra credit in their psychology classes for participation, which may have influenced those who chose to participate. Additionally, because all participants were seeking a college education, this population is overrepresented in the study compared to the general public. Generalizability is limited by the sample consisting of 83% female participants. Therefore, the findings should not be applied to male athletes. Although not as extreme, the sample was predominantly white (60%), and therefore limits applicability to other races. Importantly, because none of the constructs were manipulated or examined longitudinally, we are unable to draw conclusions with respect to the direction of these predictive analyses. Further, since mediation should be tested at three time points, and cross-sectional data were used for the mediation models, we cannot draw causal conclusions and there is the potential for substantial bias when extrapolating to longitudinal estimates (Cole and Maxwell, 2003; Maxwell and Cole, 2007). Additionally, the current study relied on a single-measure approach (Stoeber and Madigan, 2016, advocate for a multi-measure approach). Lastly, the observed associations may have been affected by the COVID-19 pandemic. For example, Uroh and Adewunmi (2021) found that some athletes in individual sports and athletes with lower athletic identity were more prone to psychological distress during the pandemic. Further, Pété et al. (2022) found that athletes who relied on avoidant coping experienced the most psychological and social distress.

### Future directions

To improve the generalizability of the current findings, research should consider different groups with a more diverse range of participants with respect to gender, race, and age. As noted above, the sample was predominantly white and female. Gender differences have been observed with respect to the association between perfectionism and coping mechanisms (Park et al., 2010). Furthermore, racial and cultural differences have been observed in perfectionism and its associations with psychological well-being (DiBartolo and Rendón, 2012). Given that gender, race, and culture influence variables examined in this study, it will be important to expand the scope of this research and consider how these processes might vary (e.g., different parental and societal expectations based on gender and race). With respect to age, future work could include adults who had participated in college athletics or children and adolescents who currently engage in athletics. By varying age groups, researchers could add a developmental lens to this work. For example, because many of these constructs are developed in childhood, incorporating aspects of developmental psychology, such as orchid versus dandelion children, would allow for the examination of risk and resiliency (Boyce and Ellis, 2005). Future research may also consider other relevant psychological factors such as locus of control and conscientiousness. Prior research has shown that perfectionists have higher levels internal locus of control compared to non-perfectionist individuals and that conscientiousness predicts increases in perfectionism over time (Periasamy and Ashby, 2002; Stoeber et al., 2009).

While prior research has been conducted on athletes, many researchers have not compared athletes directly to other groups, which may provide additional clarifications to the associations between the study constructs within different populations. Therefore, it may be beneficial to apply the same constructs to different domains of competition and rigor such as the arts, academics, and the workplace (Piotrowski, 2019; Spagnoli et al., 2021). In this way, one could examine whether differing levels of perfectionism and emotion regulation strategies are evident in different high achieving individuals and whether perfectionism is similarly related to emotion regulation and self-evaluations. Additionally, these different domains would be applicable to domain-specific self-esteem. In applying different operationalization of self-esteem, it is also recommended that alternate operationalization of perfectionism be examined, including contextualized measures that are domain specific (Stoeber and Madigan, 2016). In addition to alternate operationalization, it is recommended to use more inclusive scales with larger subscales to provide clearer insight into the psychological processes that individuals experience. Further, athletic achievements and performance should be measured to determine the impacts of perfectionism beyond psychological processes (e.g., Ahmed et al., 2021).

As discussed above, longitudinal research is needed to establish the predictive effects of perfectionism on self-esteem through the mediator of emotion regulation. For example, study measures would be assessed at least three time points (e.g., perfectionism at time1 → emotion regulation at time2 → self-esteem at time3). In this way, the direction of influence could be more firmly ascertained. For example, the research could examine if self-esteem predicts changes in perfectionism over time and/or if perfectionism predicts changes in self-esteem over time. One could also examine these variables in a more ecologically valid way. For example, emotion regulation techniques could be examined in the context of real-life events such as performative successes and failures. This would also allow for examination of the process of emotion regulation in terms of what is attended to, how situations are appraised, and what responses are made (Gross and Thompson, 2007). These emotion regulation processes could then in turn be examined in how they do or do not relate to changes in overall self-evaluations.

Additionally, experiments where random assignment or manipulation occurs should be conducted to determine causality and directionality in the data. For example, athletes or other high achieving individuals such as honors students could take part in a study designed to teach adaptive emotion regulation and coping techniques to deal with failures and potentially threatening situations. In this way, one could examine effects on self-evaluations and the links between perfectionism and self-evaluations for those who do or do not receive emotion regulation training. One could also attempt to experimentally influence perfectionistic concerns via training with coaches and/or parents to modify coach/parental expectations, reactions, and modeling. One could then observe effects on emotion regulation and self-evaluations.

## Conclusion

Examining individual differences in emotion regulation techniques within perfectionism contexts provides insight into the coping techniques and well-being of athletes. The current research specifically examined the role of maladaptive emotion regulation techniques in the relation between perfectionism and self-esteem. It was shown that both socially prescribed and self-oriented perfectionism were indirectly related to lower self-liking and self-competence through the maladaptive emotion regulation techniques of self-blame and catastrophizing. Thus, future research is warranted regarding the pathways by which perfectionism and well-being are connected, with the aim of improving people’s emotion regulation and overall psychological well-being.

## Data availability statement

The datasets presented in this study can be found in online repositories. The names of the repository/repositories and accession number(s) can be found below: https://osf.io/r54fq/?view_only=b2d35d9ac59041a0b0d0aa05f7081de7.

## Ethics statement

The studies involving humans were approved by University of North Florida Institutional Review Board. The studies were conducted in accordance with the local legislation and institutional requirements. The participants provided their written informed consent to participate in this study.

## Author contributions

HM: Writing – original draft, Writing – review & editing. MR: Writing – original draft, Writing – review & editing. PF: Writing – review & editing.

## Footnotes

^1^ https://osf.io/r54fq/?view_only=b2d35d9ac59041a0b0d0aa05f7081de7
